# Rewilding the tropics, and other conservation translocations strategies in the tropical Asia-Pacific region

**DOI:** 10.1002/ece3.1287

**Published:** 2014-10-30

**Authors:** Julien Louys, Richard T Corlett, Gilbert J Price, Stuart Hawkins, Philip J Piper

**Affiliations:** 1 Department of Archaeology and Natural History, School of Culture, History and Languages, ANU College of Asia and the Pacific, Australian National UniversityCanberra, ACT, 0200, Australia; 2 Center for Integrative Conservation, Xishuangbanna Tropical Botanical Garden, Chinese Academy of SciencesYunnan, 666303, China; 3 School of Earth Sciences, The University of QueenslandBrisbane, Qld, 4072, Australia; 4 School of Archaeology and Anthropology, Australian National UniversityCanberra, ACT, 0200, Australia

**Keywords:** Australia, conservation, extinction, mammal, Pleistocene, Southeast Asia, tortoise

## Abstract

Alarm over the prospects for survival of species in a rapidly changing world has encouraged discussion of translocation conservation strategies that move beyond the focus of ‘at-risk’ species. These approaches consider larger spatial and temporal scales than customary, with the aim of recreating functioning ecosystems through a combination of large-scale ecological restoration and species introductions. The term ‘rewilding’ has come to apply to this large-scale ecosystem restoration program. While reintroductions of species within their historical ranges have become standard conservation tools, introductions within known paleontological ranges—but outside historical ranges—are more controversial, as is the use of taxon substitutions for extinct species. Here, we consider possible conservation translocations for nine large-bodied taxa in tropical Asia-Pacific. We consider the entire spectrum of conservation translocation strategies as defined by the IUCN in addition to rewilding. The taxa considered are spread across diverse taxonomic and ecological spectra and all are listed as ‘endangered’ or ‘critically endangered’ by the IUCN in our region of study. They all have a written and fossil record that is sufficient to assess past changes in range, as well as ecological and environmental preferences, and the reasons for their decline, and they have all suffered massive range restrictions since the late Pleistocene. General principles, problems, and benefits of translocation strategies are reviewed as case studies. These allowed us to develop a conservation translocation matrix, with taxa scored for risk, benefit, and feasibility. Comparisons between taxa across this matrix indicated that orangutans, tapirs, Tasmanian devils, and perhaps tortoises are the most viable taxa for translocations. However, overall the case studies revealed a need for more data and research for all taxa, and their ecological and environmental needs. Rewilding the Asian-Pacific tropics remains a controversial conservation strategy, and would be difficult in what is largely a highly fragmented area geographically.

## Introduction

Conservation translocations are increasingly being discussed as a viable tool for the conservation of species, populations, and ecosystems in response to threats caused by loss of habitats and reductions in their quality, biological invasions, and the predicted future impacts of climate change (IUCN/SSC 2013). Established conservation translocation strategies range across a spectrum from relatively low-risk population reinforcements, where conspecifics already exist in potential release sites, to relatively high-risk releases of ecological replacements for globally extinct taxa (IUCN/SSC 2013). Most contentious have been attempts to extend the baseline for reconstituting ecosystems back into the late Pleistocene, before the apparently concentrated episode of ‘megafaunal’ extinctions: losses of large vertebrates that appear to mark the arrival of modern humans in many parts of the world (Donlan et al. 2006). ‘Pleistocene rewilding’ markedly extends the concept of translocations by regarding taxa that have been regionally extinct for millennia as indigenous, and by a willingness to introduce ecological replacements for such extirpated forms where necessary. Given the conservation issues triggered by invasive aliens, and the general failure to predict such problems in advance from species traits, good arguments can be made for extreme caution when considering ecological replacement as a conservation strategy (Rubenstein et al. 2006; Caro and Sherman 2009; Oliveira-Santos and Fernandez 2010). On the other hand, such translocations have already produced some documented conservation successes (Griffiths et al. 2011, 2013; Gross 2013) and will undoubtedly continue to be considered as possible solutions for biodiversity conservation.

Rewilding strategies expand on established IUCN guidelines by an emphasis on continent-scale conservation, enabled by a focus on large, connected, protected core areas and a motivation to embrace species introductions for the purposes of ecosystem function restoration (Sandom et al. 2013). Rewilding programs have received considerable attention in Eurasia and North America (e.g., Donlan et al. 2005; Martin 2005; Zimov et al. 2012; Gross 2013), but much less so in tropical regions where continued human population growth, rapid land-use change, and uncontrolled exploitation of natural resources have combined to create unprecedented threats to some of the most diverse environments in the world (Sodhi and Brook 2006; Laurance et al. 2011). Our study examines the possibility and limitations of conservation translocations in the Asia-Pacific region, concentrating on tropical Southeast Asia, Australia, and the Pacific islands. We consider the full spectrum of conservation translocation strategies for a subset of relatively large-bodied vertebrate faunas with which we are familiar, and assess conservation goals ranging from species conservation to ecosystem function restoration. This study is not exhaustive; neither does it outline all the political, logistical, and ecological problems confronting translocation strategies in the Asia-Pacific. Rather we examine specific case studies in order to derive some general conclusions and highlight the potentials and pitfalls for conservation translocations, and in particular rewilding, in the most endangered biodiversity hotspot in the world (Duckworth et al. 2012).

## Methods

### Terminology

We follow the IUCN/SSC's (2013) terminology with respect to conservation translocation, summarized as follows: (1) *Reinforcements* refer to the translocation of organisms into a release area where conspecifics are already present. The primary conservation aim of reinforcements is to enhance population viability. (2) *Reintroductions* refer to the translocation of an organism into an area that it had previously occupied as part of its indigenous range, but where it has since disappeared. The primary conservation aim of reintroduction is to re-establish a species in its indigenous range, and is inclusive of goals seeking to perform an ecological function. The IUCN/SSC defines the indigenous range of a species as “the known or inferred distribution generated from historical (written or verbal) records, or physical evidence of the species’ occurrence” (IUCN/SSC 2013, p.2). In the following case studies, we distinguish between historical and physical (in our study, specifically palaeontological or archaeological) records, given that the latter can extend a species’ occurrence millions of years before present, in geographies radically different from what is found today, and might refer to non-analogous or disharmonious assemblages (sympatric associations in the past which are now allopatric; e.g., Lundelius 1989; Price 2004; Medway 1972). (3) *Assisted colonization* refers to the release of an organism outside its indigenous range for the explicit purpose of saving that organism from extinction. (4) *Ecological replacement* refers to the release of an organism outside its indigenous range to perform a specific ecological function. The IUCN further indicates that such replacements will often involve congeneric species, and we differentiate between closely related replacements (i.e., congeneric species) and distantly related replacements. We also use the term *rewilding*, and follow the Sandom et al. (2013) definition of this term, namely that it refers to continent-scale conservation with three basic criteria: translocation into large, protected core areas, appropriate connectivity between these areas, and the translocation of organisms for the purposes of restoring ecosystem functioning. Its primary conservation goals are mitigating anthropogenic ecosystem impacts (Sandom et al. 2013, p. 432). Pleistocene rewilding seeks to restore ecosystems to pre-human conditions, and its main underlying assumption is that humans were responsible for the extinction of many large-bodied organisms in the late Pleistocene (Donlan et al. 2006).

**Figure 1:**
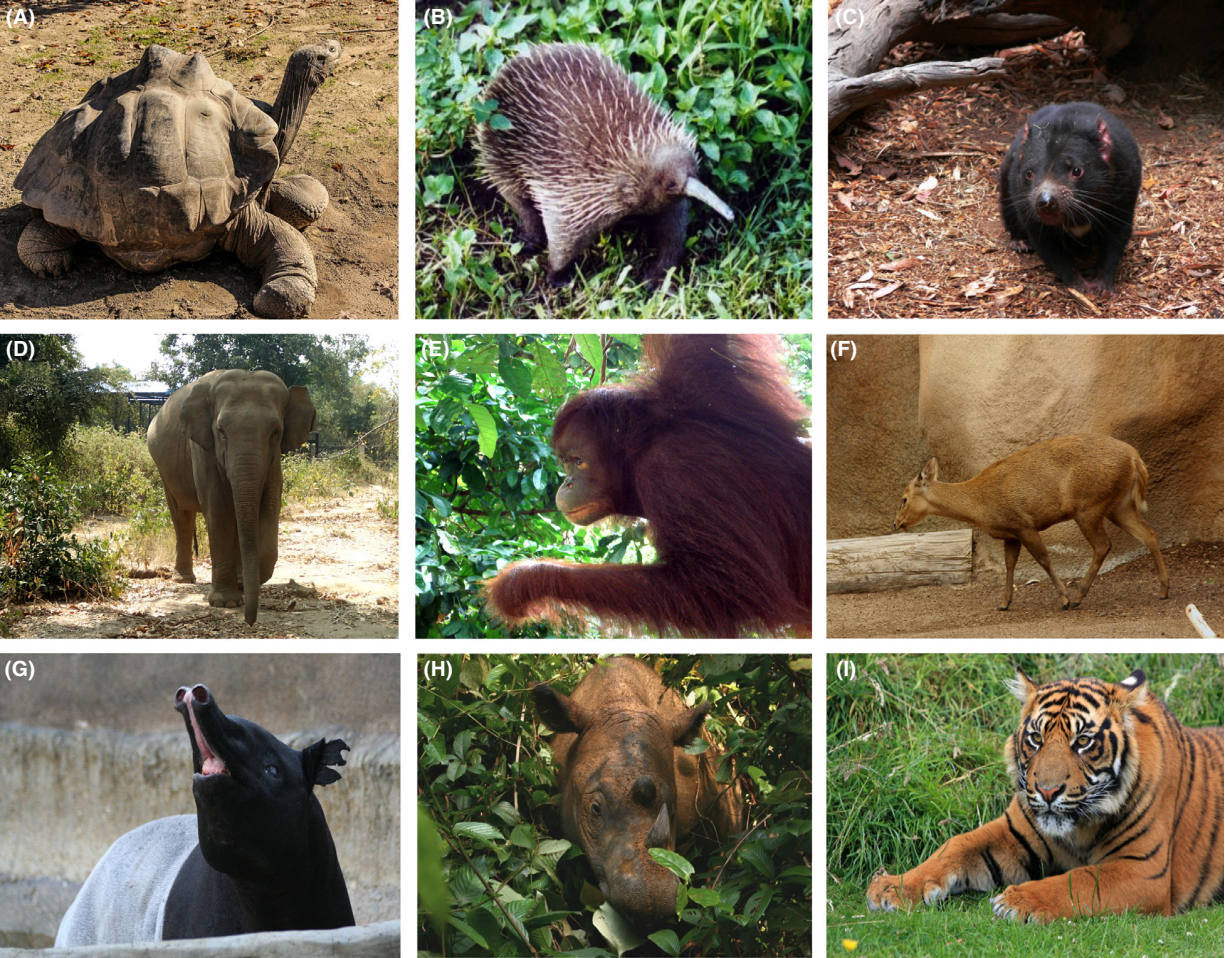
Examples of the nine taxa considered; (A) tortoise; (B) long-beaked echidna; (C) Tasmanian devil; (D) Asian elephant; (E) Bornean orangutan; (F) Calamian hog deer; (G) Malayan tapir; (H) Sumatran rhino; (I) tiger. (Photo credits: (A) J. DeMeres; (B) Jaganath; (C) L. Frerichs; (D) J. Louys; (E) G. Louys; (F) S. Hanko; (G) Sepht; (H) W. v Strein; (I) K. Arnold; photo sources: (A) http://pixabay.com; (B), (C), (F), (G), (H) http://wikipedia.com; (D), (E) personal collection; (I) http://publicdomainpictures.net).

We considered only relatively large, vertebrate taxa with a fossil record, as these are the taxa we are most familiar with and because we wished to include an assessment of Pleistocene rewilding strategies for this region. Unfortunately, other potentially endangered species, such as plants, invertebrates, and fungi have a very limited to non-existent fossil record in this region, and thus are not considered here. Within our study group, we further narrowed our case studies to one reptile and eight mammals. The selected taxa met the following criteria: they are listed as “endangered” or “critically endangered” by the IUCN in our region of study; they have a written and fossil record that is sufficient to assess past changes in range, as well as ecological and environmental preferences, and the reasons for their decline; and they have suffered massive geographical range restrictions since the late Pleistocene (for examples of range reduction maps, see Antoine 2012; Louys 2012). These organisms are spread across diverse taxonomic and ecological spectra and have the potential to be translocated within former ranges or to act as species substitutions for conservation and/or ecological restoration purposes (Fig. 1). Taxonomic authority for each species follows the IUCN (see Table 1 for taxon-relevant references). Most exhibit a unique ecological role within their ecosystems, but this was not a criterion for selection as we also wished to examine reinforcements and reintroductions. The taxonomic resolution of our selection varies from the species to family level (Table 1).

**Table 1:** Summary list of taxa considered for tropical rewilding, with descriptions of the factors considered in the case studies

Taxon	Previous range	Current range	Taxonomy	IUCN status and reference	Habitat	Ecological role	Reason for decline	Available stock	Costs/Risks	Benefits	Priority sites	Other comments
Giant tortoises	Mainland Asia to Fiji	None	Meiolaniidae (several extinct fossil species endemic to multiple island groups/regions)	Extinct	Wide variety, rainforest to grassland and woodland	Seed dispersal, maintain vegetation heterogeneity	Hunting, invasive mammals, climate change	Taxon substitutions available from Galapagos Islands	May spread invasive plants, need to control rats	Partial ecological restoration, ecotourism	Uninhabited Pacific islands	Indian island reintroductions successful
Long-beaked echidna	New Guinea, mainland Australia	New Guinea	*Zaglossus bruijnii, Zaglossu sbartoni, Zaglossus attenboroughi*	All species critically endangered (Leary et al. 2008a,b,c) (Leary et al. 2008a,b,c)	Subalpine, rain forest	Soil turnover, invertebrate feeder	Hunting and habitat loss	Very limited populations in zoos and New Guinea	None known	Species security	Protected Australian tropical rainforest	
Tasmanian devil	Mainland Australia, Tasmania	Tasmania	*Sarcophilus harrisii*, 1 or 2 fossil species	Endangered (Hawkins et al. 2008)	Forest, moorlands, grass/woodlands	Scavenger with hunting ability	Hunting, disease, climate change, vehicles	Successful captive breeding programs, zoos		Species security, suppress feral carnivores	Mainland Australia	Captive breeding programs already successful on mainland
Elephants and stegodons	China to Timor	China, India, Borneo	*Elephas maximus*, multiple extinct species of *Elephas, Palaeoloxodon,* and *Stegodon*	Endangered and extinct (Choudhury et al. 2008)	Forest and grasslands	Seed dispersal, maintain vegetation heterogeneity	Hunting, habitat loss, climate change	Many captive elephants available	Human-elephant conflict	Dispersal of megafaunal fruits, ecotourism, national pride	Large protected forest areas in Holocene range	Individual elephants successfully released in several areas
Orangutans	South China to Java	Borneo and Sumatra	*Pongo abelii* *Pongo pygmaeus* unknown fossil spp.	*Pongo abelii* critically endangered; *Pongo pygmaeus* endangered (Ancrenaz et al. 2008; Singleton et al. 2008)	Forests, including degraded areas	Seed dispersal	Habitat loss, hunting, climate change	Breed well in captivity, captured from deforested areas and confiscated pets	Human conflict	Ecotourism, seed dispersal, species security, individual welfare	Large protected areas in Borneo, Sumatra, maybe Peninsular Malaysia	Small scale reintroductions in historic range already underway.
Calamian hog deer	Palawan	Calamianes Islands	*Axis calamianensis*	Endangered (Oliver et al. 2008)	Grasslands, open woodland and secondary forest	Browser, maintenance of environmental heterogeneity	Hunting, human settlement and agricultural expansion	Limited wild populations	Human predation	Species security, game animal	Protected areas in Palawan	
Tapir	South China to Java	Indochina	*Tapirus indicus*	Endangered (Lynam et al. 2008)	Primary and secondary forest	Browsing/seed dispersal	Loss of habitat, hunting	Bred in captivity	Human predation	Species security	Borneo	
Rhinoceroses	South China to Sundaand Luzon	India, Indochina, Java, Sumatra Borneo	*Rhinoceros unicornis, Rhinoceros sondaicus, Dicerorhinus sumatrensis*	*Rhinoceros unicornis*vulnerable (extinct from region considered); *Rhinoceros sondaicus* criticallyendangered; *Dicerorhinus sumatrensis* critically endangered (van Strien et al. 2008a,b; Talukdar et al. 2008)	Grasslands, primary forest	Grazers, browsers, seed dispersal	Hunting,	Indian available, Sumatran, Javan probably not	Possible human-wildlife conflict	Species security, ecological restoration	Well- protected forest	
Tiger	Mainland Asia to Bali	Siberia, Sumatra, India, Indochina	*Panther atigris* 3 subspecies extinct	Endangered (Chundawat et al. 2011)	Mixed grass/woodland, rain forest	Apex predator	Hunting	Breeds well in captivity	Human-tiger conflict, need large prey populations	Restoration of predation, national pride, ecotourism	Large protected areas in SE Asia	Successful reintroduction in India

From the case studies, we assessed the potential prospects for translocations and the most significant challenges facing the translocation success of each taxon. We distilled these potentials and pitfalls into a conservation translocation matrix (Table 2). Specifically, we examined three criteria: risks, benefits, and feasibility. Within each of these criteria, we asked a number of questions derived from our case studies. For risk, we examined the type of translocation strategy available. For population restoration, we considered reinforcements less risky than reintroductions. Likewise for conservation introductions, we considered release of organisms into historical ranges less risky than into fossil ranges. Closely related ecological replacement was considered more risky again, but less risky than distantly related ecological replacement. We scored taxa as to whether their translocation could cause harm to human populations. Finally, we examined the ease with which translocated organisms could be monitored or removed if necessary. For feasibility, we looked at whether the conservation threat to the organism was known, and whether it had been removed; whether the organism could subsist in degraded or altered environments; whether stock was available; and finally whether community support was already present. For benefit, we examined whether conservation was targeted at the taxon level, or whether it would potentially affect an entire ecosystem. Where taxa spanned more than one answer, scores within these were averaged. The conservation scores were then plotted on a ternary graph and the positions of the taxa on this graph compared.

**Table 2:** Conservation matrix. Each axis represents a ranked summary of the factors considered in each case study, with weightings assigned according to the key. The maximum values for each axis are listed

Axis	Key	Giant tortoise	Long-beaked echidna	Tasmanian devil	Elephant	Orangutan	Calamian hog deer	Tapir	Rhinoceros	Tiger	Max
Feasibility											
Threat	0 = unknown; 1 = known but still present; 2 = known and removed	2	2	1	1	1	1	1	1	1	2
Ecology well understood	0 = no; 1 = yes	0	0	1	1	1	1	1	0	1	1
Can survive in degraded habitat	0 = no; 1 = yes	1	0	1	1	1	0	1	1	1	1
Available stock	0 = no; 1 = yes	1	0	1	1	1	1	1	0	1	1
Community support already present	0 = no; 1 = yes	0	0	1	0	1	0	0	0	0	1
Feasibility Total		4	2	5	4	5	3	4	2	4	6
Risk											
Population restoration	0 = no; 1 = reinforcement; 2 = reintroduction	0	2	1.5	1.5	1.5	2	1.5	1.5	1.5	2
Conservation introduction	0 = no; 1 = historical range; 2 = fossil range; 3 = ecological replacement closely related; 4 = ecological replacement distantly related	4	2	2	2.5	1.5	2	1.5	2	1.5	4
Poses risk to human population	0 = no; 1 = yes	0	0	0	1	0	0	0	0	1	1
Ease of control/eradication	0 = easy; 1 = difficult/unkown	0	0	1	1	1	1	1	1	1	1
Risk total		4	4	4.5	6	4	5	4	4.5	5	8
Benefit											
Species conservation (highest)	0 = extinct; 1 = endangered; 2 = critically endangered	0	2	1	1	2	1	1	2	1	2
Ecosystem function	0 = no/unknown; 1 = regional; 2 = continental	1	1	2	2	2	1	2	2	2	2
Benefit total		1	3	3	3	4	2	3	4	3	4

### Case studies

#### Giant tortoises (Testudinidae and Meiolaniidae)

During the Quaternary, giant tortoises inhabited the Asia-Pacific from mainland Asia to Australia and as far as Fiji, but are now extinct from the region (Hansen et al. 2010). The Quaternary range of the Testudinidae in our region of study included parts of Wallacea, the Philippines, and the Ryukyu Islands, as well as continental Asia (Turtle Taxonomy Working Group 2011). Giant tortoises from Sahul (Australia and Papua New Guinea) and the Pacific were from a different, now extinct, family Meiolaniidae (Gaffney 1991; White et al. 2010). Regardless of taxonomic affiliation, humans and introduced invasive mammals appear to have been responsible for giant tortoise extinctions in some parts of the Pacific (Van Denburgh 1914; White et al. 2010) and islands within Wallacea (Morwood and Van Oosterzee 2006). Today, giant tortoises typically occur in dense populations on ungulate- and predator-free remote islands such as Aldabra atoll in the Indian Ocean (Hamilton and Coe 1982), and are broad-diet herbivores, frugivores, and omnivores with highly adaptive digestive systems (Bonin et al. 2006; McMaster and Downs 2008). They occupy a range of vegetation types from coastal shrublands and dry deserts to rainforests (Hansen et al. 2008; Pedrono 2008). Their widespread distribution suggests that they are capable of adapting too many types of environments, and are particularly suited to isolated insular environments. Their extinction from island communities has resulted in severely unbalanced biotic communities and the loss of some ecosystem functions (Swingland and Klemens 1989; Griffiths et al. 2010).

Conservation translocation of tortoises has been recommended because they are considered low-risk, easy to breed and regulate, and have versatile feeding behaviors (Griffiths et al. 2010; Hansen et al. 2010). They are considered keystone species in many ecosystems, acting as important seed dispersers (Hansen et al. 2008; Jerozolimski et al. 2009), and creating and maintaining habitat heterogeneity by trampling or digging burrows (Means 2006). Tortoise taxon substitutions can contribute significantly to ecotourism, as on privately-owned tourist islands in the Seychelles (Hansen et al. 2010), and giant tortoise translocation has been successfully implemented on Indian Ocean islands (Samways et al. 2010; Griffiths et al. 2011). In Vanuatu, faunal diversity has declined since initial human arrival (Steadman 2006), with giant tortoises (*?Meiolaniadamelipi*) becoming extinct within the last 3000 years (White et al. 2010). Although it is not known precisely what impacts these changes had on ecosystems, it appears that vegetation became more open and disturbed in some parts of Vanuatu following extinctions (Hope and Spriggs 1982; Wirrmann et al. 2011), and a significant decline in ecological diversity due to invasive mammals and the loss of giant tortoises seems probable.

The Galapagos and Aldabra Islands have similar tropical environments to Vanuatu, both being extensive volcanic and limestone archipelagos with habitats including disturbed open secondary vegetation and grassland, montane and lowland rainforest, and coastal mangrove vegetation (Hamann 1979; Gibson and Hamilton 1983; Mueller-Dombois and Fosberg 1998). The Aldabra giant tortoise (*Aldabrachelys gigantea*) feeds in mixed inland and coastal scrub/grass (Gibson and Hamilton 1983), while the Galapagos giant tortoise (*Chelonoidis nigra*) occupies a wide range of habitats, migrating seasonally between lowland grasslands and elevated woods and scrub (De Vries 1984). Thus one of these tortoise species might be considered for ecological replacement and there are a number of uninhabited islands in the Vanuatu Archipelago that could act as suitable translocation sites. The isolated nature of these islands suggests that a tortoise introduction could not be considered as rewilding, as viable ecological connections between islands would probably only happen with direct human intervention. Quarantining the tortoises before relocation would reduce the chance of introducing exotic plant species from seeds in their guts (Hansen et al. 2010). Exotic predators, such as rats, may have to be controlled, although juvenile tortoises bred in captivity and released would have a good chance of surviving.

Translocation potential: ecological replacement

#### Long-beaked echidnas (*Zaglossus* spp.)

The fossil record of long-beaked echidnas indicates a wide geographical distribution in the late Quaternary, with fossils recovered from across the Australian mainland (Price and Webb 2006; Helgen et al. 2012) and throughout New Guinea (Sutton et al. 2009). Today, extant species of *Zaglossus* are reliably known only from New Guinea, where until recently they occurred over much of the island (Flannery 1995). A *Zaglossus* specimen apparently collected in the Kimberly region in 1901 was recently recognized in the collections of the British Museum of Natural History, leaving open the possibility that long-beaked echidnas (*Z. bruijnii*) may still be extant in northwestern Australia (Helgen et al. 2012). Three species of long-beaked echidna are currently recognized in New Guinea: *Z. bruijnii*, *Z. bartoni*, and *Z. attenboroughi*. They occur in rainforest and subalpine regions, and occupy a unique feeding niche in New Guinea, being specialists on earthworms and subterranean arthropods (Griffiths et al. 1991). All are apparently threatened by hunting and habitat loss (Flannery and Groves 1998).

Although some Australian marsupials target subterranean arthropods and worms (e.g., some bandicoots), none currently occupies an identical niche to *Zaglossus*. Habitat potentially suitable for supporting long-beaked echidnas is present across northern Australia, and they could be introduced to that region (Helgen et al. 2012). Relaxation of hunting pressure would be critical for the establishment of introduced populations. Although introductions of long-beaked echidnas are unlikely to have major ecological benefits for Australian ecosystems, establishing new populations would contribute to species security and would perhaps have beneficial regional effects. The small home ranges and ease of tracking of the short-beaked echidna (Augee et al. 1975) suggests monitoring of introduced populations would be straightforward. The biggest challenge is the availability of individuals for translocations. *Zaglossus bruijnii* has not been recorded in the wild since the 1980s and *Z. attenboroughi* is known only from one specimen collected in 1961 (although ethnographic evidence suggests that they are still relatively common; Baillie et al. 2009). *Z. bartoni*occurs occurs in low populations (Opiang 2009).

Translocation potential: reintroduction (outside historical range, within fossil range)

#### Tasmanian devil (*Sarcophilus harrissi*)

The Tasmanian devil once occurred throughout the Australian mainland but is now restricted to Tasmania. It is the largest extant marsupial carnivore and a specialized carrion-feeder, inhabiting a variety of environments, including moorlands, coastal heath, sclerophyll forests, forestry plantations and cleared pasturelands (Jones 2008). Modern devils reach their greatest densities in environments with mixed patches of grassland and woodland, and apparently avoid tall or dense wet forests (Jones and Barmuta 2000). In contrast, fossil records from northern mainland Australia suggest that they once inhabited tropical rainforests (Horton 1977; Hocknull 2005). Mainland extinction of the devil has been attributed to various factors, including competition from the introduced dingo since c. 4000 BP, human land-use intensification, innovations in hunting technologies (Johnson and Wroe 2003), and enhanced climate variation from around 6000 BP (Brown 2006). Significant declines in devil populations in Tasmania during historic times have resulted from hunting (Owen and Pemberton 2011). Today, the greatest threats to devils are vehicle strikes and disease, principally the Devil Facial Tumor Disease. Populations have declined by 60% in the last decade, with current estimates of 10–20,000 individuals remaining (Buckley et al. 2012). Suggested conservation strategies include the culling of diseased populations, with subsequent reinforcement from disease-free stock. Current breeding programs include zoos and dedicated reserves both in Tasmania and on the Australian mainland, with the principal aims being the reintroduction of devils back to their historic Tasmanian range (Lunney et al. 2008).

There are many habitats across northern tropical Australia (and most of mainland Australia) that could potentially support populations, including both closed and open woodlands, and mixed grasslands west of the Great Dividing Range. Significantly, climate has not changed drastically since the time of their mainland extirpation, and is unlikely to cause detrimental impacts on reintroduced populations. We would predict that with the relaxation of direct indigenous and European hunting pressure, the chances of successful devil reintroductions would be enhanced, as was the case where koalas were inadvertently “reintroduced” to parts of their former range (Price 2012) with unanticipated and explosive population growth (Masters et al. 2004). However, an ongoing threat to reintroduced devils might come from competition with dogs, including both dingoes and feral domesticates introduced after European colonization. An additional potential threat could be through ingestion of the poisonous cane toad (*Rhinella marina*), introduced to northern Australia in the 1930s. Their impacts on northern quolls (*Dasyurus hallucatus*), closely related to devils, have been catastrophic (Shine 2010), and potential impacts on devils could be equally devastating. However, relatively small-bodied taxa, such as those similar in size to cane toads, do not typically form a significant component of the diet of modern Tasmanian devils in Tasmania today, with their preferred food choice being medium to large-bodied herbivorous marsupials such as wallabies (Jones and Barmuta 2002). A clearer understanding of the potential impacts of cane toads and of devil-dog interactions is vital to the establishment of populations on mainland Australia. In addition to enhancing species security, reintroducing devils may help control alien predators such as cats, in turn implicated in the extinctions of small-bodied marsupials across mainland Australia (Johnson et al. 2007; Lazenby and Dickman 2013).

Translocation potential: reinforcement, reintroduction (outside historical range, within fossil range)

#### Elephants (Elephantidae)

Proboscideans (elephants and stegodons) were ubiquitous in Pleistocene Asia, occurring from China, throughout tropical continental Asia, to the continental islands of Borneo, Sumatra and Java, and the oceanic islands of Luzon, Mindanao, Sulawesi, Flores, Sumba, Timor, and Sangihe (van den Bergh et al. 2001a,b). Elephants and stegodons apparently coexisted over much of this range, although only stegodons inhabited the smaller islands. There were at least five species of proboscidean present in the late Pleistocene, but by the Holocene only the Asian elephant (*Elephas maximus*) survived, confined to mainland Asia, Sumatra and Java (Louys et al. 2007; Louys 2012). Asian elephant distributions have become progressively restricted in the last two millennia. The reasons for local and regional extinctions are unclear, but Asian elephants have been hunted for thousands of years and island populations of proboscideans may have been vulnerable to early hominins and/or geological disturbance (i.e., volcanic eruptions; van den Bergh et al. 2009). Asian elephants require access to forest, although they will feed in the open. They have unique roles in forest ecology, including long-distance seed dispersal of large “megafaunal fruits” (Campos-Arceiz and Blake 2011; Sekar and Sukumar 2013) and the modification of vegetation structure through browsing and trampling (Corlett 2013). Although the prolonged coexistence of stegodons with elephants at many sites suggests that they cannot have been complete ecological equivalents, their size-related ecological roles are likely to have been similar.

Asian elephants could potentially be reintroduced to any large forests within their Holocene range, and their introduction as taxon substitutes on islands previously inhabited by stegodons in the late Pleistocene might be comparable to introductions of Testudinidae as ecological replacement for now extinct Meiolaniidae. Reintroductions of elephants to protected forests, either on mainland SE Asia or on smaller, environmentally degraded islands would increase species security, restore seed-dispersal and other ecological services, enhance the welfare of individual animals, and act as an ecotourism attraction. It has also been suggested that elephants could be introduced to Australia to act as ecological replacements for the extinct megafauna (Bowman 2012), but this would be both hugely controversial and of unclear benefits. While elephants do not breed well in captivity, thousands of surplus elephants currently exist in captivity in Asia (Taylor and Poole 1998). Problem elephants are routinely captured and moved in several areas (Fernando et al. 2012), captive elephants are also released within their historical range for welfare reasons (Corlett, personal observations), and a small population of feral elephants is established outside their native range in the Andaman Islands. Moreover, the existing population on Borneo may be feral animals of Javan origin (Cranbrook et al. 2007). The potential for elephants to pose a risk to human populations and communities (e.g., Zhang and Wang 2003) would need to be evaluated prior to any translocations.

Translocation potential: reinforcement, reintroduction (historical and fossil ranges), assisted colonization, ecological replacement, rewilding

#### Orangutans (*Pongo* spp.)

In late Pleistocene Asia orangutans were widespread, from around 30°N in southern China, throughout continental Southeast Asia to Sumatra, Borneo and Java. Holocene records, however, are confined to Borneo and Sumatra (Ibrahim et al. 2013), and by historical times orangutans were restricted to dense rainforests with few human inhabitants. Although some authors have attributed this dramatic range loss to environmental changes (Louys et al. 2007; Ibrahim et al. 2013), orangutans certainly were hunted from the late Pleistocene onwards, and large, slow-breeding animals are expected to be particularly vulnerable to extirpation (Corlett 2007). The Bornean (*P. pygmaeus)* and Sumatran (*P. abelii*) orangutans are currently considered endangered and critically endangered, respectively, by the IUCN, with habitat loss, hunting and the pet trade the major threats (Rijksen and Meijaard 1999; Ellis et al. 2006). Orangutans were historically confined to dense lowland and lower-montane rainforests, but their ability to persist in degraded landscapes suggests a greater range of habitat tolerance, which is also consistent with their wide Pleistocene distribution (Ibrahim et al. 2013). Orangutans prefer fruit when it is available, but can subsist on a variety of less nutritious plants foods (Galdikas 1988). The seeds in most fruits they consume are dispersed, and the orangutans’ large size, strength, and tree-climbing probably make them particularly important for the dispersal of tree species with large-seeded fruits (Corlett 2009).

Orangutans could be reintroduced to any large forests within their late Pleistocene range, including logged and degraded forests, with Borneo and Sumatra having priority, followed by Peninsular Malaysia. Reintroductions to protected forests could help save the species from extinction, as well as restoring seed-dispersal services, enhancing the welfare of individual animals, and acting as an ecotourism attraction. Animals for reintroduction are currently available from captures in areas undergoing deforestation and from confiscated pets, and both species breed well in captivity. The successes of previous small-scale reintroductions within their historical ranges remain unclear (Russon 2009).

Translocation potential: Translocation potential: reinforcement, reintroduction (historical and fossil ranges)

#### Rhinoceroses (Rhinocerotidae)

Asian rhinos are members of the Rhinocerotidae, although they fall within two genera and comprise three species. The Javan (*Rhinoceros sondaicus*) and Sumatran (*Dicerorhinus sumatraensis*) rhinos are relatively common in fossil deposits of Quaternary age throughout Southeast Asia, and were present throughout the region well into the late Holocene through to historical times (Antoine 2012). Over the last 200 years, their ranges and populations have dramatically declined due to habitat loss and extensive hunting, a practice that continues today (Milliken et al. 2009; Antoine 2012). Since its extinction in Vietnam (Platt 2011), the Javan rhino is now restricted to a tiny (∼40 individuals) population in west Java, and is probably the most endangered large-bodied mammal in the world. The Sumatran rhino is currently only found as scattered, tiny populations in Sumatra and Borneo. The Indian rhino (*Rhinoceros unicornis*) was widespread in Pleistocene China and in mainland Indochina and Java in the early and middle Pleistocene (Antoine 2012). It is currently restricted to India, Nepal and Bhutan, and is extinct in the region we are examining (Southeast Asia). Javan and Sumatran rhinos are both considered critically endangered, and Indian rhinos are vulnerable. All rhinos have the same broad habitat requirements, evidenced by their co-occurrence in several fossil sites in Southeast Asia (e.g., Duoi U'oi, Ban Fa Suai), although some niche partitioning between them would necessarily exist. Indian rhinos are grazers and commonly inhabit grasslands. Little is known of the ecology of the Javan rhino, although its last population currently lives in lowland tropical rainforest. Sumatran rhinos are smaller than Javan or Indian rhinos, and are currently found in tropical rainforests, cloud forests and montane moss-forest, as well as occasionally being observed at forest margins and in secondary forests (Nowak 1999). All three species are implicated in the dispersal of large-seeded fruits (Corlett 2007).

Large areas of suitable habitat for reintroductions occur through the previous ranges of all three species. There have even been highly controversial suggestions of introducing rhinos into Australia as a substitute for the extinct megafauna (Bowman 2012). Reintroductions of African black rhinoceroses into North Luangwa National Park, Zambia were successful (van der Westhuizen et al. 2010), and seemingly successful reintroductions of the Indian rhinoceros occurred in India (Sinha 2011). The biggest problems are the lack of surplus individuals in the wild or captivity, and the continued threat from hunters. Realistically only Indian rhino populations are present in sufficient numbers to consider reintroduction. As with elephants, potential damage to crops and agricultural land would need to be evaluated before reintroduction.

Translocation potential: reinforcement, reintroduction (historical and fossil ranges), assisted colonization, ecological replacement, rewilding

#### Malayan tapir (*Tapirus indicus*)

The Malayan tapir (*Tapirus indicus*) is the last remaining member of the perissodactyl family Tapiridae inhabiting the Old World. Palaeontological and archaeological records indicate that the Malayan tapir was once distributed throughout Southeast Asia from Myanmar in the west to China south of the Qinling Mountains in the east and as far south as Java (Cranbrook and Piper 2013). There have been a few historic reports of their presence in southern Vietnam, Cambodia and Laos (Grubb 2005), but Malayan tapirs now appear to be exclusively restricted to Sumatra, Peninsular Malaysia, parts of southern Myanmar, and southwestern and peninsular Thailand (Linkie et al. 2013). The IUCN considers the species to be endangered with continuing population declines.

The archaeological record supports anecdotal historical evidence for the presence of the Malayan tapir on Borneo in the recent past, and Piper and Cranbrook (2007a) suggested the conservation areas of natural lowland forest at Binyo-Penyilam and Bukit Sarang within the Planted Forest Zone in Sarawak, Malaysian Borneo would be suitable for reintroductions. The two conservation areas cover a total of c. 40,000 ha and are linked by an additional 20,000 ha in the Bukit Mina wildlife. The enclosed and connected nature of the reserves would be suitable for the management of a rewilding strategy. The demise of the tapir in Borneo appears related to human predation (culminating in the rhino hunts of the 1930s) rather than incompatible changes in environment during the Holocene. Historically, Malayan tapirs inhabited lowland tropical evergreen rainforests and riverine valleys, particularly edge habitats, swampy tracts and disturbed jungle. Contemporary tapirs can tolerate almost all types of degraded habitat, and even relatively close proximity to human populations if left unmolested. They play a key role in ecosystem maintenance, seed predation and dispersal, selective browsing and forest gap retention (Medici et al. 2008). Tapirs have mild temperaments and pose no threats to human populations, although they might cause some damage to crops. The IUCN Tapir Specialist Group has noted that tapirs are highly adaptable to changes in diet and different environmental conditions and can overcome some of the greatest challenges presented to reintroduced animals (Medici et al. 2008). The greatest threat to tapirs (especially adults) is hunting, although they are currently not favored prey for hunters (Corlett 2007; Linkie et al. 2013). Zoo breeding has been relatively successful (Ryder, Medway 1983) and captive animals could be used in release programs.

Translocation potential: reinforcement, reintroduction (historical and fossil ranges), rewilding

#### Calamian hog deer (*Axis calamianensis*)

The Calamian hog deer is endemic to the Philippines and is currently found only on the Calamianes Islands of Busuanga, Calauit, and Culion, between Palawan and Luzon (Corbet and Hill 1992). There are no contemporary or historical records of large deer taxa on Palawan, but archaeological research in the north and central regions of the island has identified the past presence of what is almost certainly the Calamian hog deer, with skeletal remains identified throughout the early and mid-Holocene, until c. 4000–3000 years ago (Piper et al. 2008; Ochoa and Piper in press).

The preferred habitats of the Calamian hog deer are grasslands, open woodland and secondary forest regrowth (Oliver et al. 2008). Palaeoenvironments of Palawan suggest that similar types of habitat would have been widespread during the Pleistocene (Wurster et al. 2010) prior to the expansion of tropical rainforests and coastal inundation (including island splitting), concomitant with climatic amelioration at the end of the last glacial period. This, coupled with increased hunting pressure from expanding human populations, probably resulted in the local extinction of hog deer (Ochoa and Piper in press). The Calamianes Islands are northeast of the Sundaic tropical rainforest zone and have retained seasonal grassland and open woodland throughout the Holocene, and this has perhaps been a key factor in the prolonged existence of hog deer on these smaller islands. Some protected but partially deforested areas of Palawan might provide potential habitats for the reintroduction of the Calamian hog deer. Reintroduction of the Calamian hog deer is unlikely to have any impacts on the Palawan bearded pig (*Sus ahoenobarbus*)*,* the only large-bodied surviving endemic mammal. Deer populations might have economic importance in terms of providing game to hunt. There are no large predators on Palawan and the only threat to adult animals would be human predation.

Translocation potential: reinforcement, reintroduction (historical and fossil ranges)

#### Tiger (*Panthera tigris*)

Tigers were present from China, the Philippines through to Sunda throughout the Quaternary (Piper and Cranbrook 2007b; Piper et al. 2008; Louys 2012, 2014) but their range has dramatically reduced during historical times as a result of hunting and habitat destruction. Today they occupy only 7% of their former range and have been eliminated from Bali, Java, Borneo, and southern China (Sanderson et al. 2006; Walston et al. 2010). Tigers occupy a wide range of environments, from taiga and temperate forests to lowland tropical rainforests. The isolation of Southeast Asian subspecies is a recent phenomenon (Louys 2012, 2014) and their predominant occurrence in rainforests may be more a result of historical and biogeographic factors, rather than habitat preferences (Kitchener and Dugmore 2000).

Reintroductions of this species will likely be motivated by national and local pride and a widespread belief that the relatively secure captive populations are no substitute for free-living populations. Tigers breed well in captivity so the availability of animals would not limit reintroduction efforts, particularly if the minor distinctions between the tropical subspecies were ignored. The major limitations are the potential for human-tiger conflicts and the need for large areas with sufficient large prey (Brietenmoser et al. 2009; Johnsingh and Madhusudan 2009). While reintroductions would restore the tiger's role as apex predator and potentially limit overpopulation of pigs and deer, their preferred prey (Hayward et al. 2012), human hunters already keep these populations at low densities almost everywhere in the region (Corlett 2007).

Translocation potential: reinforcement, reintroduction (historical and fossil ranges), rewilding

### Results

**Figure 2:**
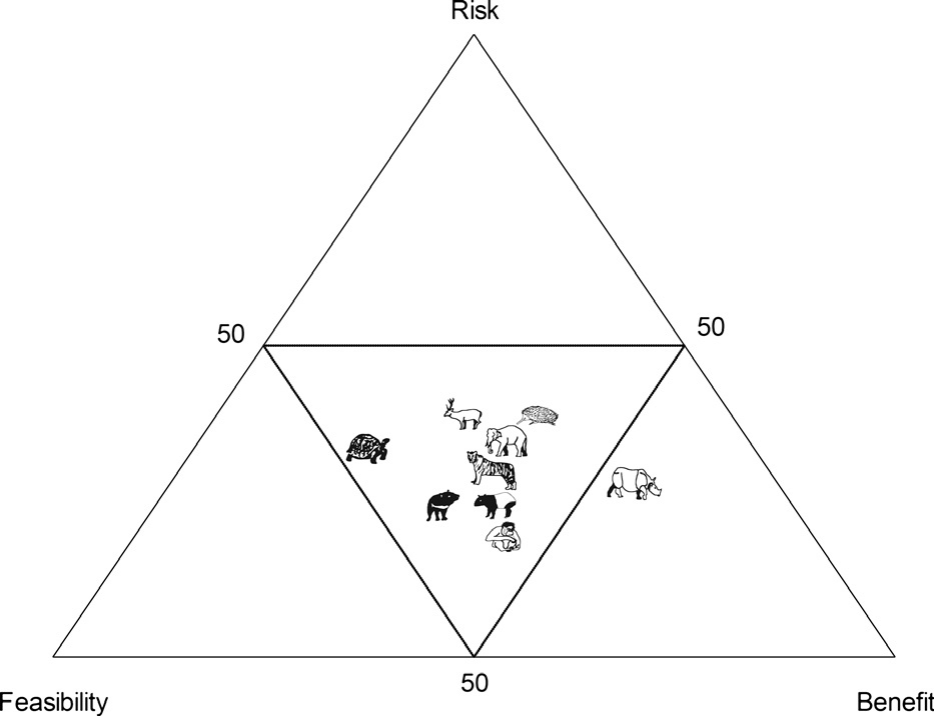
Ternary graph illustrating the relative positions of each taxon considered in the case studies and scored according to the criteria listed in Table 2. Ideally, species suitable for conservation translocation should be situated as close to the base of the outer triangle; and a species with equal feasibility and benefit would be situated at the apex of the inner triangle.

Amongst the taxa we examined, the ones with the lowest risk for conservation translocation, with respect to feasibility and benefit, are the orangutans, Tasmanian devils, and tapirs, in that order (Fig. 2). Conservation translocation of orangutans and tapirs were judged to be of more conservation benefit but lower feasibility than the Tasmanian devil. The devil's feasibility would be increased if it were introduced Australia-wide, rather than just in tropical northern Australia – the only region we explicitly considered in this study. Tortoises are also highly feasible for conservation translocation, although their risk factor is increased because this would represent a distantly related introduction of an ecological equivalent. Rhinoceroses had the highest conservation benefit with respect to feasibility and risk, as they are potential candidates for both rewilding and reintroductions, and two of the three species examined are critically endangered. On the basis of the conservation translocation matrix and ternary diagram (Fig. 2), orangutans, Tasmanian devils, tapirs, and tortoises are highlighted as the taxa with the greatest potential for future conservation translocation.

## Discussion

The principal obstacle for rewilding the Asia-Pacific tropics is the need for multiple, large, connected, conservation areas. While this might be feasible for parts of continental Southeast Asia, and some of the larger islands in the region (e.g., Borneo), rewilding, as defined by Sandom et al. (2013), would not be possible for smaller islands as connections between them would necessitate human intervention. The reintroduction of the Tasmanian devil throughout Australia might also be considered a case of rewilding, and is perhaps the least controversial and most feasible of the case studies examined here. Sandom et al. (2013) argue strongly that one of the main drivers behind the push for rewilding is the extinction of megafauna during the late Pleistocene. In the Americas, it has been argued that the late Pleistocene represents the last time that humans had limited or no impacts on ecosystems, so that this period is a reasonable baseline for determining the indigenous range of a species and identifying sites for reintroductions (Martin 2005; Sandom et al. 2013). Human colonization of Australia occurred toward the end of the late Pleistocene, although the impacts of humans on tropical Australian ecosystems remain controversial and poorly understood (Bird et al. 2013; Wroe et al. 2013). In the Pacific, human arrival on islands has had dramatic impacts on island ecosystems during the last few millennia (White et al. 2010). The argument for the initial timing of human impacts is less straightforward in Southeast Asia, where there was probably continuous occupation by hominins from the early Pleistocene to the present (Louys and Turner 2012). While the region suffered a significant number of extinctions, a direct human role in mainland and island Southeast Asian extinctions in the Pleistocene (including through environmental degradation), though plausible, is not strongly supported by current evidence (Louys et al. 2007; Corlett 2013). What is clear is that since the end of the Pleistocene, people have had a considerable impact on those species that managed to persist into the Holocene through both hunting and environmental modification, including those taxa covered in our case studies.

One issue with Pleistocene rewilding is that early and mid-Pleistocene environments were often very different from more recent ones and may not provide suitable baselines for ecosystem states. Even late Pleistocene environments in the Asia-Pacific were radically different from the present, particularly during the last glacial maximum, when low sea-levels resulted in the connection of many present-day islands; temperatures and, in many places, rainfall were lower; and multiple lines of evidence indicate a greater extent of open and semi-open habitats (Louys and Meijaard 2010; Price 2013; Reeves et al. 2013). The late Pleistocene ranges of many taxa may therefore include areas that subsequent environmental changes have made unsuitable for reintroduction. On the other hand, the more recent—Holocene—ranges of most of the taxa we consider have been reduced by hunting and habitat destruction, and so greatly underestimate potential modern ranges. Moreover, open and semi-open habitats, albeit anthropogenic, are again widespread. The potential effects of climate change on potential release sites will also need to be seriously examined (Thomas 2011). These problems argue for the use of the entire historical record, from the last interglacial to the present day, but judged carefully and on a taxon-by-taxon basis, as evidence for past and potential future habitats, rather than a broad-brush approach when considering Pleistocene rewilding or reintroductions.

The taxa included in our case studies are mostly associated with forests, although some can persist in more open habitats (Table 1). Importantly for the availability of sites for reintroduction, several taxa (elephants, Rood et al. 2010; rhinos, Nowak 1999; orangutans, Husson et al. 2009; tapirs, Cranbrook and Piper 2013; Tasmanian devils, Jones 2008; and tigers, Rayan and Mohamad 2009) are known to survive, and in rare cases thrive, in disturbed and degraded forests. In Southeast Asia, the most extensive areas of degraded lowland forest have been subject to selective logging, often through multiple cycles. Despite its massive impacts on forest structure, logging appears to have a relatively small effect on many forest animals (e.g., birds, dung beetles and by inference, mammals; Edwards et al. 2011), so a mosaic of logged and unlogged areas is likely to provide a suitable habitat for reintroductions. These areas will still need to be protected from hunters, however.

The case studies cover all the major dietary specializations, from herbivory (including grazing, browsing and frugivory), to invertebrate consumption, predation on living vertebrates, and scavenging. In several cases, the taxa considered are known to have had unique roles in their ecosystems which cannot be substituted by surviving species: for example, elephants, and probably rhinoceroses and orangutans, as dispersers of seeds in very large “megafaunal” fruits (Campos-Arceiz and Blake 2011), and tigers as apex predators (Corlett 2011). Giant tortoises probably had a unique seed dispersal role on oceanic islands (Blake et al. 2012). The niche of the Tasmanian devil has been at least partly filled by introduced feral predators, but there are indications that their presence can significantly suppress cat numbers (Lazenby and Dickman 2013). Several taxa are also known or inferred to have impacted vegetation structure and habitat heterogeneity through their feeding and trampling activities (proboscideans, rhinoceroses, tapirs, tortoises, and probably *Zaglossus*) (e.g., Corlett 2013).

The introduction of elephants and rhinos into Australia has been argued on the basis of the ecological role that they may fill(Bowman 2012), specifically those left vacant by now-extinct Pleistocene megafauna (Hall and Walter 2014). While we do not consider these introductions feasible or even desirable, an interesting analogous introduction has already taken place in Australia. The banteng (*Bos javanicus*) was introduced in northern Australia in 1849, and since then the herd in the Garig Gunak Barlu National Park in the Northern Territory is the world's largest wild population of this endangered species (Brook et al. 2006). The conservation paradox presented by this species is detailed by Brook et al. (2006), and ranges on a spectrum from whether this species should be considered a feral pest that has no place in a national park, to its presence in the park considered a conservation refuge for a species endangered in its indigenous range. While the situation with the banteng differs from the case studies examined here because it was introduced to Australia over a century ago, it does highlight how difficult it may be to effectively manage an introduced large-bodied species within a national park over the longer term.

The restoration of ecological roles is a major potential benefit of conservation introduction in most cases (Table 2). Other benefits include reducing extinction risk in endangered species by establishing new populations (most taxa) and supporting ecotourism (tortoises, orangutans, and potentially elephants). National pride is a strong motivation for at least the elephant, tapir and tiger, but could also be nurtured for other taxa. The major risks concern human-wildlife conflict. Risks of harm to people and domestic animals, and crop damage, are likely to limit opportunities for reintroducing elephants and tigers, and to a lesser extent orangutans and rhinoceroses, even to existing protected areas. These risks might be reduced by appropriate fencing, but such barriers are expensive to erect and require regular maintenance. Illegal hunting is likely to be a major threat to populations, particularly for high-value species such as tigers and rhinoceroses, and such threats must be eliminated before reintroductions are considered (Johnsingh and Madhusudan 2009).

The other major limiting factor is availability of stock for reintroduction. Surplus domestic elephants are available following the decline in their economic role and wild elephants are being translocated from sites undergoing clearance (Corlett, personal observations). Orangutans are available from confiscated illegal pets and captures in clearance sites (Russon 2009). Tigers, tapirs, and devils breed well in captivity, but Javan and Sumatran rhinoceroses and *Zaglossus* have no surplus captive animals. All giant tortoises from the region are now extinct, but taxon substitutes are available (Griffiths et al. 2013). Limitations on the availability of animals for reintroduction and/or the area of habitat available may give rise to demographic or genetic problems in the future, necessitating continued monitoring and additional releases (e.g., Russon 2009).

## Conclusions

There is undoubtedly potential for conservation translocation in the tropical Asia-Pacific. No taxa we considered would be considered too risky for reintroduction on the basis of our scoring system, and the higher risk to benefit and feasibility scores some taxa received was due more to a lack of data rather actual risk. This largely reflects the conservative approach we took when selecting taxa for our case studies. For example, we did not consider the introduction of African hyenas into Southeast Asia as an ecological replacement for the wide-ranging, Pleistocene SE Asian hyenid *Pliocrocuta perrieri*. Additional studies of all taxa are clearly needed before any translocations take place. Nevertheless, the Malayan tapir, Asian elephant, Indian rhino, tiger, Tasmanian devil, Calamian hog deer, and orangutans have existing populations that make them feasible targets. These species have experienced significant range reductions within the Holocene, and areas where they used to occur could potentially be used for reintroduced populations. Among these taxa, the tapir, devil, Calamian hog deer and orangutan are least likely to be involved in significant human-wildlife conflict, while tigers and elephants would require very large areas or expensive fencing. *Zaglossus* and the Sumatran and Javan rhinos do not have existing source populations, although the former might conceivably be found in sufficient numbers with increased levels of exploration in New Guinea. Ecological replacement is a controversial conservation technique, but the apparently successful introduction of exotic tortoises on islands in the Indian Ocean suggests that it is a viable option for at least these large, slow-moving, slow-breeding, and easily relocatable animals. Rewilding the tropics might be feasible for parts of continental Southeast Asia, however significant obstacles remain, particularly regarding human-animal conflict and control of reintroduced taxa. On the basis of our case studies and the conservation matrix we constructed, we recommend that tapirs, orangutans, tortoises, and devils should be targeted for more detailed studies, followed, if still supported, by reversible experimental translocation into suitable habitats.
